# Reproducibility-Based Field Validation of Complementary Subsurface Sensing Using a Quantum Diamond Magnetometer and Multi-Frequency Ground-Penetrating Radar

**DOI:** 10.3390/s26175439

**Published:** 2026-08-28

**Authors:** Chang-Geun Oh, Byunghoon Choi, Dong-Hoon Shin

**Affiliations:** 1 Department of Aerospace Industrial and Systems Engineering, Hanseo University, Taean 32158, Republic of Korea; 2 Department of Energy Resources Engineering, Inha University, Incheon 22212, Republic of Korea; bhoon1121@inha.edu; 3 Department of Civil and Construction Engineering, Kangwon National University, Samcheok 25913, Republic of Korea; donghoonshin@kangwon.ac.kr

**Keywords:** quantum diamond magnetometer, nitrogen-vacancy (NV) center, ground-penetrating radar, subsurface sensing, sensor complementarity, reinforced concrete inspection, field reproducibility, ferromagnetic anomaly detection

## Abstract

**Highlights:**

**What are the main findings?**

- NV-diamond QDM detected magnetic responses associated with buried ferromagnetic targets under realistic pavement conditions.
- QDM and multi-frequency GPR provided complementary magnetic and dielectric information across different pavement structures.

**What are the implications of the main findings?**

- QDM can complement GPR where subsurface ferromagnetic features are of interest.
- Combined sensing may improve subsurface characterization of structurally complex pavement environments.

**Abstract:**

Underground infrastructure inspection requires sensing methods capable of characterizing both ferromagnetic and non-ferromagnetic subsurface features. This study evaluated a portable nitrogen-vacancy (NV) quantum diamond magnetometer (QDM) together with multi-frequency ground-penetrating radar (GPR; 250, 500, and 800 MHz) at a 90 m full-scale pavement test site containing documented subsurface targets. Repeated stationary QDM measurements were first conducted to characterize temporal variability and establish site-specific magnetic baselines, followed by a 1 m interval spatial survey and corresponding GPR measurements. The QDM showed reproducible spatial magnetic patterns despite substantial variability in reinforcement-dense sections, and all 10 cross-session measurements remained within ±1.5 standard deviations of their repeated-measurement baselines. Ferromagnetic targets produced localized magnetic responses, whereas post hoc classification using disturbance-normalized magnetic anomalies showed limited target-level discrimination (AUC = 0.495). GPR likewise showed limited post hoc target classification (500 MHz AUC = 0.472), but section-level radar responses differed significantly across pavement environments. The two modalities exhibited contrasting section-level responses while showing weak and inconsistent point-level correspondence. These results indicate that NV-diamond magnetometry and GPR provide complementary physical information, while repeated magnetic measurements are important for interpreting QDM observations under heterogeneous field conditions.

## 1. Introduction

### 1.1. Background

The inspection of underground infrastructure has become increasingly important because aging transportation systems, buried utilities, and reinforced concrete structures are continuously exposed to deterioration caused by environmental loading and long-term service conditions. Undetected subsurface conditions, including buried metallic objects, hidden structural components, pavement deterioration, loose zones, and voids, may contribute to structural failures and sinkhole formation [1]. Consequently, reliable non-destructive inspection technologies capable of characterizing both metallic and non-metallic subsurface features are important for infrastructure maintenance.

Ground-penetrating radar (GPR) is widely used for subsurface inspection because it provides rapid, continuous imaging without excavation [2]. Multi-frequency GPR can estimate pavement thickness, identify layer interfaces, detect voids, and visualize underground utilities through contrasts in dielectric permittivity [3]. However, its performance depends strongly on the electromagnetic properties and structural complexity of the subsurface. Signal attenuation, multiple reflections, and clutter from reinforced concrete can complicate interpretation under certain pavement conditions.

Magnetic sensing provides physically distinct information because ferromagnetic materials generate disturbances in the Earth’s magnetic field. Conventional systems, including fluxgate, proton-precession, and optically pumped magnetometers, have been used for archaeological investigation, unexploded-ordnance detection [4], and geological exploration [5]. These technologies have different practical trade-offs: fluxgate sensors provide compact vector measurements but are susceptible to offset and drift; proton-precession magnetometers provide stable scalar measurements but generally have lower sampling rates; and optically pumped magnetometers can achieve high sensitivity but may impose instrument-specific operating-range and environmental constraints.

Recent advances in quantum sensing [6] have enabled the development of quantum diamond magnetometers (QDMs) based on nitrogen-vacancy (NV) centers in diamond [7]. NV-diamond magnetometry provides solid-state, room-temperature vector magnetic-field measurement without cryogenic cooling [8,9,10,11,12]. However, these characteristics do not establish superior field performance relative to conventional magnetometers. Because the present experiment did not include side-by-side measurements with conventional magnetic sensors, it was not designed to establish comparative advantages in sensitivity, signal-to-noise ratio, measurement error, accuracy, or dynamic range. A recent review identified reinforced-concrete screening as a prospective rather than established application of quantum magnetometry and highlighted the limited field validation available under realistic infrastructure conditions [13]. Table 1 therefore summarizes the principal strengths and limitations of representative magnetometer technologies and their relevance to complementary subsurface sensing with GPR.

### 1.2. Previous GPR–Magnetometry Studies and Research Gap

Previous studies have extensively investigated the integrated or comparative use of GPR and magnetic sensing for subsurface characterization. In archaeological applications, Chianese et al. combined magnetic mapping, GPR, and magnetic-susceptibility measurements to characterize buried remains, while Matera et al. integrated two GPR systems with high-resolution magnetometry to enhance subsurface imaging [14,15]. Schneidhofer et al. evaluated GPR and magnetometry interpretations against archaeological excavation results, and Turner et al. compared GPR, magnetic gradiometry, and electromagnetic induction for buried-feature characterization [16,17]. Similar approaches have been applied to buried-object and unexploded-ordnance detection [18] and joint airborne GPR–magnetometer systems for landmine detection [19]. Collectively, these studies establish that combining GPR and magnetometry is not in itself novel; rather, the modalities provide complementary information because GPR primarily responds to dielectric properties and subsurface geometry, whereas magnetometry responds to magnetic materials and local magnetic-field disturbances. However, previous combined studies have predominantly employed conventional magnetic sensors, while field evidence involving NV-diamond magnetometry remains limited.

Against this background, the relevance of NV-diamond magnetometry is not that magnetic sensing is new to GPR-based subsurface investigation, but that it provides a distinct solid-state, room-temperature, vector-capable implementation. Its potential benefits for integrated field sensing must nevertheless be considered alongside environmental variability, acquisition time, and the current maturity of the technology (Table 1). Accordingly, the specific contribution of this study is the field-scale evaluation of a portable NV-diamond magnetometer alongside multi-frequency GPR at a controlled pavement test site with documented subsurface conditions, together with repeated magnetic measurements to characterize field reproducibility and site-specific baseline variability. The study therefore examines whether spatial magnetic responses remain interpretable relative to temporal field variability under structurally heterogeneous pavement conditions without assuming superiority over conventional magnetometers.

### 1.3. Research Objectives and Contributions

A repeated-measurement approach was first used to characterize QDM field reproducibility and establish a site-specific magnetic baseline. Spatial magnetic measurements were then compared with 250, 500, and 800 MHz GPR responses acquired over corresponding pavement sections and documented subsurface conditions. The study addresses four research questions:

1. RQ1 (Field reproducibility): What level of field measurement reproducibility can be achieved with a QDM under realistic multi-material pavement conditions, and how does the observed variability differ between structurally simple and reinforced pavement sections?
2. RQ2 (Detection boundary): Under the tested field conditions, do buried ferromagnetic targets produce distinguishable magnetic responses while the evaluated non-ferromagnetic subsurface conditions do not produce consistent localized magnetic signatures?
3. RQ3 (Baseline characterization): To what extent can repeated measurements characterize a site-specific magnetic baseline and distinguish persistent magnetic patterns from temporal variability across different pavement environments?
4. RQ4 (Complementarity with GPR): How do the sensing characteristics of a QDM and multi-frequency GPR complement one another when evaluating the same subsurface targets at the same field test site?

The main contributions are: (1) a field-scale comparative evaluation of a portable NV-diamond QDM and multi-frequency GPR at a controlled underground test site with documented subsurface conditions, and (2) a repeated-measurement framework for characterizing field reproducibility and establishing a site-specific magnetic baseline prior to spatial surveying. The resulting QDM responses are compared with multi-frequency GPR observations while explicitly distinguishing the demonstrated field complementarity from claims of superiority over conventional magnetometers.

## 2. Materials and Methods

### 2.1. Experimental Test Site

The Korea Expressway Corporation maintains a full-scale underground test site in Yesan-gun, Chungcheongnam-do, Republic of Korea. We conducted field measurements at this site to evaluate the complementary sensing characteristics of a QDM and multi-frequency GPR under realistic infrastructure conditions.

The test site consisted of three consecutive pavement sections, each approximately 30 m long, representing typical road and bridge transition environments:

(1) Asphalt concrete (ascon) pavement;
(2) Approach slab;
(3) Concrete pavement (15 m of plain concrete followed by 15 m of reinforced concrete).

**Figure 1:**
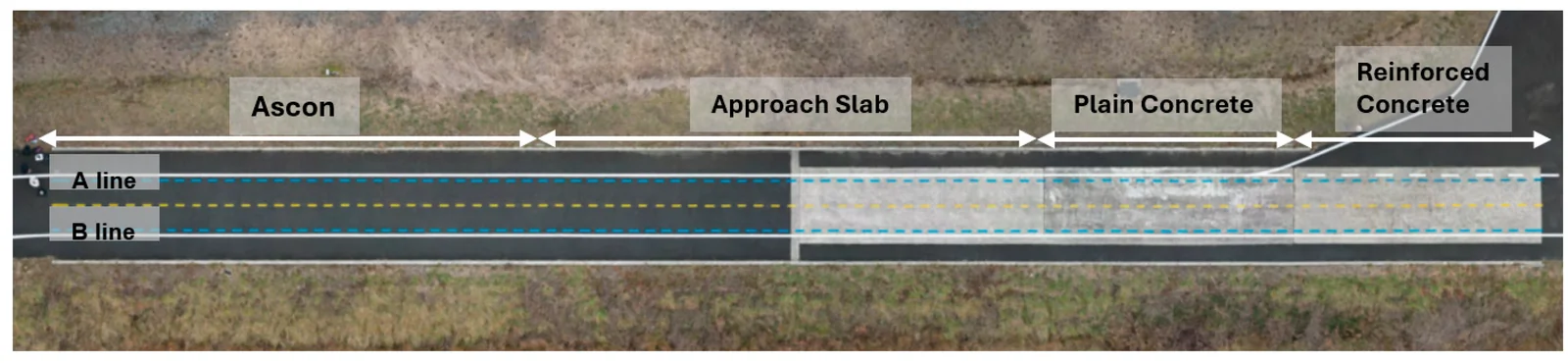
Layout of the experimental test site showing the pavement sections and survey lines.

Figure 1 illustrates the layout of the test site and the pavement sections investigated in this study. The site consisted of a single-lane roadway with shoulders on both sides. The spatial points, material types, and burial depths of the underground targets were predetermined and documented before construction. Subsurface targets were installed beneath two predefined survey lines (Lines A and B), indicated by the blue dashed lines in Figure 1. The available survey width was approximately 6.6 m, and the total survey length was 90 m. The installed subsurface targets established reliable ground truth for sensor validation. These targets included ferromagnetic steel plates, simulated loose zones, EPS voids, and reinforced concrete structures. Table 2 summarizes the buried targets, their distances from the starting point of the test site, and their burial depths. We used the engineering drawings to define the survey layout and measurement locations. After completing signal processing and initial anomaly identification, we consulted the drawings as the ground-truth reference for validation.

Unlike laboratory experiments conducted using artificial targets in homogeneous soil, the present test site embedded realistic pavement materials, reinforced concrete, and bridge transition structures, thereby providing a representative environment for field validation. In addition to the documented buried targets summarized in Table 2, the engineering records also identified localized loosened-ground zones in the ascon section, which were examined as representative non-ferromagnetic ground conditions during the field survey. The test site was located near a highway interchange, with continuous vehicle traffic on a roadway approximately 50 m from the survey area, representing a potential source of transient magnetic interference during QDM measurements.

### 2.2. Ground Truth

The test site included subsurface targets distributed across the three pavement sections to represent a variety of infrastructure inspection scenarios. The ascon section contained a buried steel plate, expanded polystyrene (EPS) blocks (Styrofoam), and simulated loose zones. The approach-slab section included deeply buried cavity-plus-steel-plate targets beneath reinforced concrete, representing a typical bridge-transition environment. The plain-concrete section contained EPS voids and an unreinforced concrete drain pipe, while the reinforced-concrete section contained additional EPS voids and an unreinforced concrete drain pipe within a reinforcement-dense pavement environment.

We did not use the ground-truth information during signal processing or anomaly identification. Instead, we consulted it only after completing the data processing to evaluate the spatial correspondence between the measured sensor responses and the known underground targets. The documented target spatial points, dimensions, material types, and burial depths served solely as ground-truth references for post hoc validation and interpretation of the experimental results and did not influence data acquisition or preprocessing.

**Figure 2:**
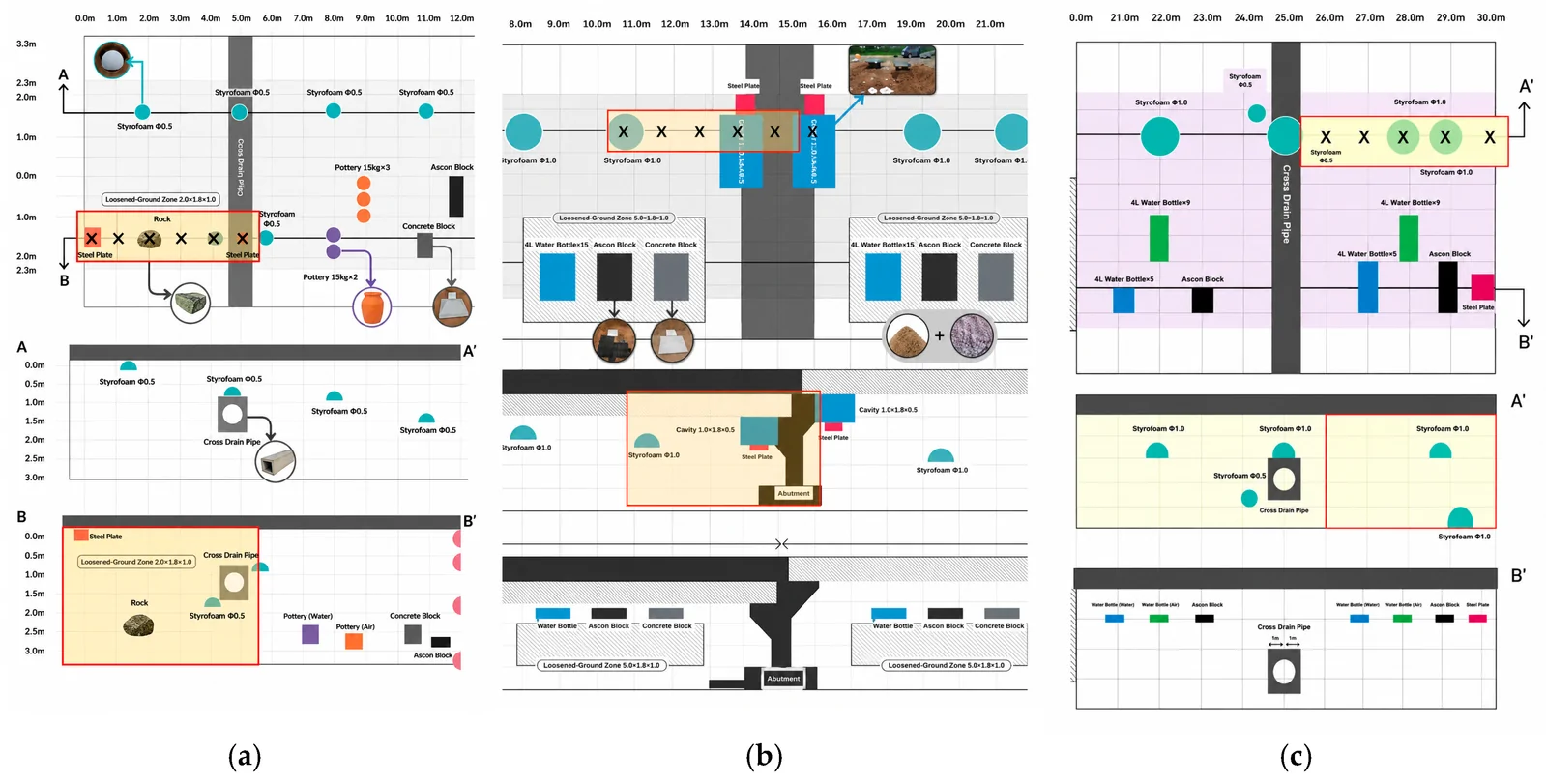
Selected measurement points (X marks) in the three pavement sections. “A” and “B” denote survey Lines A and B shown in Figure 1. The lower panels present the corresponding vertical layouts of the buried targets beneath each survey line. (**a**) Asphalt concrete (Ascon) section; (**b**) approach slab section; (**c**) reinforced concrete section.

Figure 2 presents the selected measurement points (marked by X) used in this study. Red rectangles indicate the measurement regions analyzed in this study. We chose the measurement spatial points to represent the major subsurface targets distributed across the ascon, approach slab, and reinforced concrete sections. The lower panels show the corresponding vertical layouts of the buried targets beneath survey Lines A and B, which were used as ground-truth references during the validation process.

### 2.3. Quantum Diamond Magnetometer

We performed magnetic field measurements using the SBQuantum quantum diamond magnetometer (SBQDM; SBQuantum Inc., Sherbrooke, QC, Canada). Table 3 presents the specifications of the SBQDM used in this study. The SBQDM simultaneously records the three orthogonal magnetic field components, $B_{x}$, $B_{y}$, and $B_{z}$, together with the total magnetic field magnitude, defined as

(1) $$B_{norm}=\sqrt{B_{x}^{2}+B_{y}^{2}+B_{z}^{2}}$$

**Figure 3:**
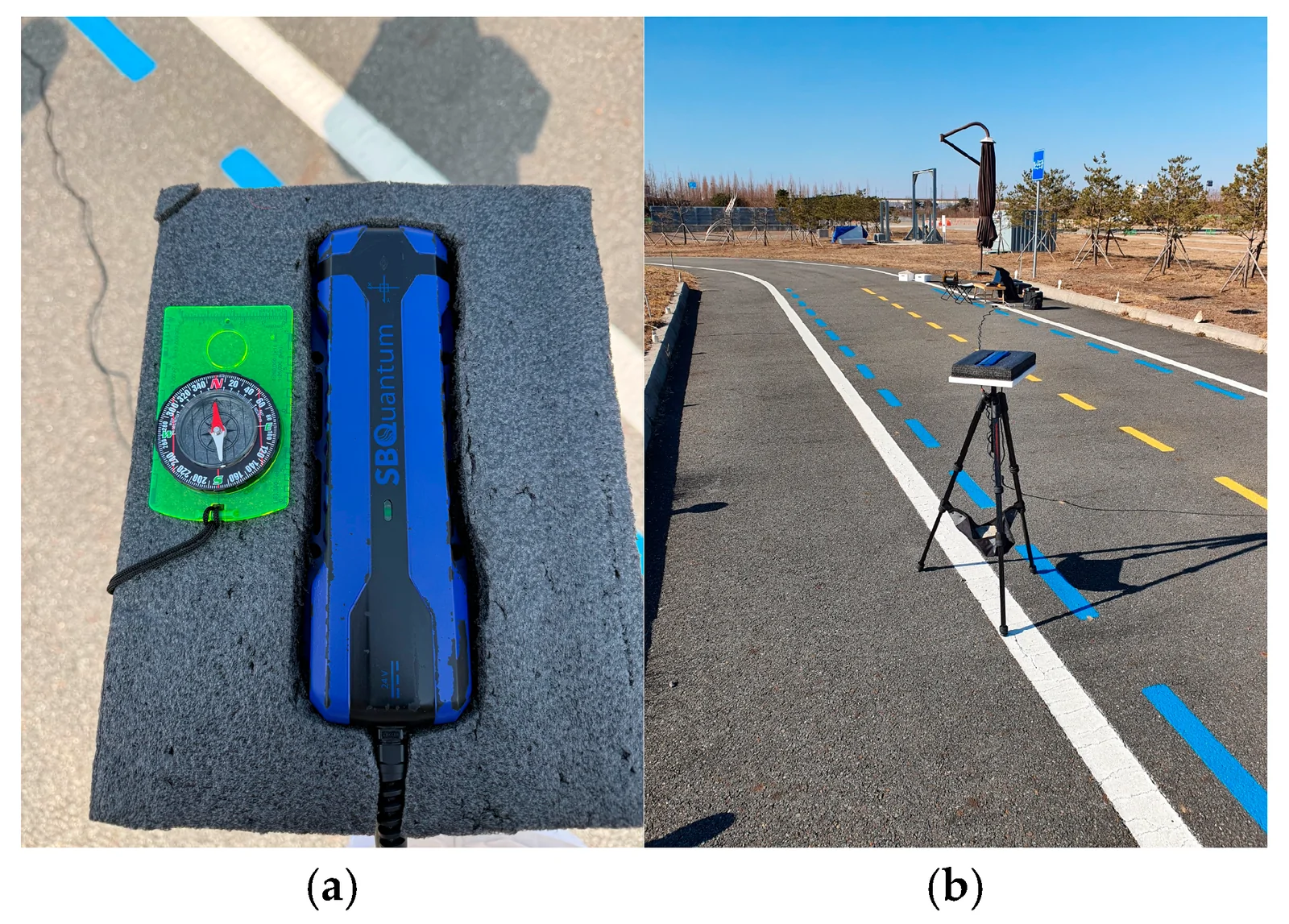
(**a**) SBQDM mounted in a custom EVA foam holder; (**b**) the tripod-based field measurement setup. The compass was used only for pre-measurement orientation to magnetic north and was not present during data acquisition.

The sensor applied its firmware-embedded calibration model automatically during operation; no separate external calibration verification was performed before or after the field measurements. We mounted the sensor on a nonmetallic carbon-fiber tripod (Manfrotto MTBFRTC4GTFB Befree GT PRO; Manfrotto, Cassola, Italy). To protect the sensor during field deployment, we mounted the SBQDM in a custom EVA foam holder, which also provided stable positioning throughout the measurements. To minimize operator-induced variability, we kept the sensor orientation (aligned with magnetic north), tripod configuration, and measurement height (50 cm) identical for all observations. Figure 3 shows the experimental sensor setup.

At each survey point, the sensor was allowed to acclimate for 16 s before data recording. Each measurement then yielded 1001 magnetic field samples acquired during an effective recording period of approximately 37 s. Based on the dense-survey acquisition timestamps, the median interval from the end of one recording to the start of the next was approximately 67 s, including repositioning and acclimation, corresponding to a median stop-and-go measurement cycle of approximately 105 s per 1 m survey point. Although the total measurement cycle varied among experimental conditions, all measurements produced the same number of samples as determined by the firmware configuration. We calculated representative magnetic field values from the stable portion of each time series after removing transient disturbances and completing the preprocessing procedure. We also performed repeated stationary measurements at selected spatial points to characterize temporal magnetic variability and establish a site-specific magnetic baseline. In addition, we conducted dense spatial surveys over the entire 90 m survey line at 1 m intervals.

### 2.4. Ground-Penetrating Radar Acquisition

**Figure 4:**
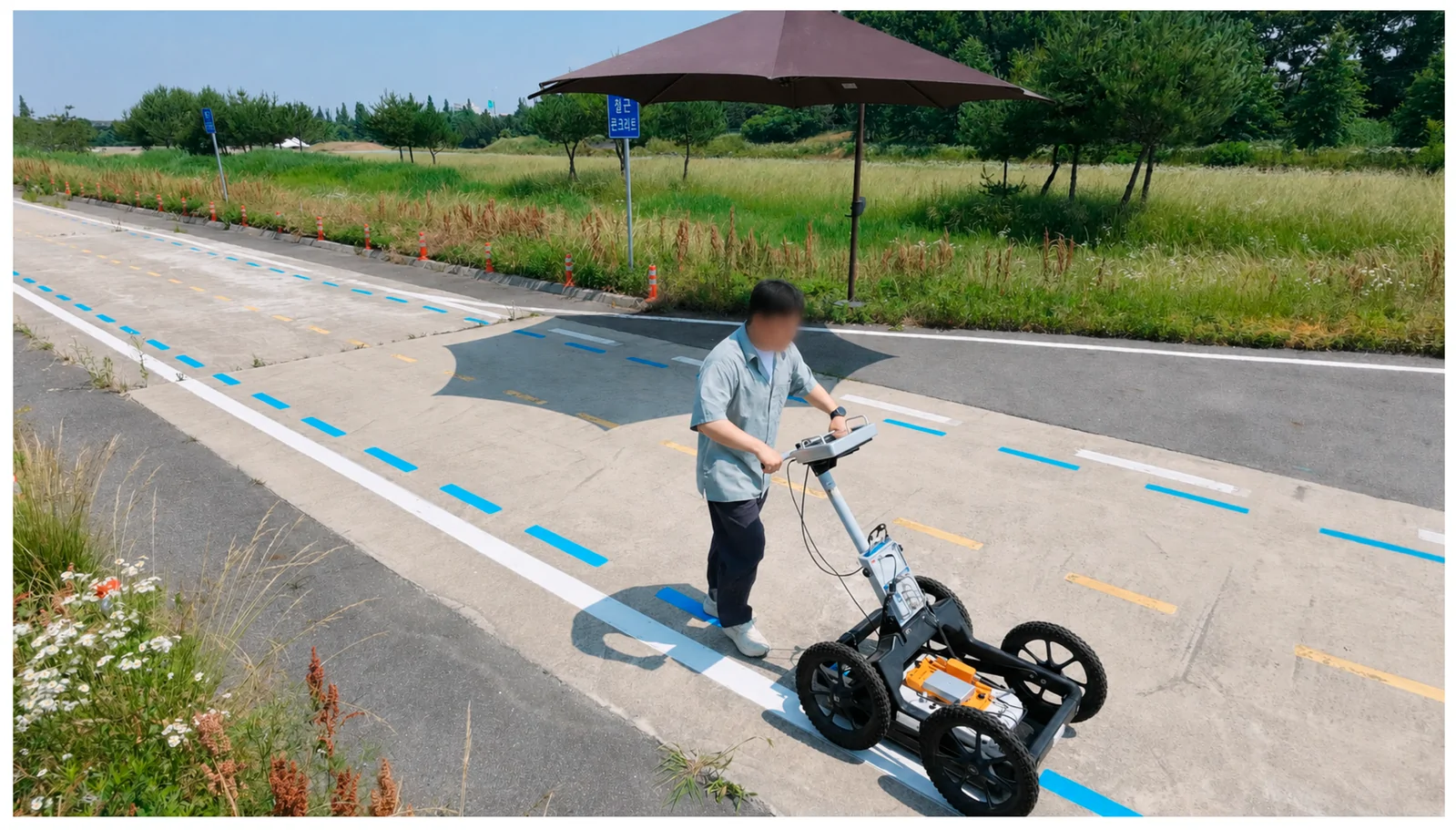
MALÅ ProEx GPR system used in the field experiments. The Korean sign in the background indicates the reinforced-concrete section.

We conducted GPR measurements using the MALÅ ProEx system (Guideline Geo AB, Sundbyberg, Sweden; Figure 4) along the same survey lines used for the QDM measurements. We used three shielded antenna frequencies (250, 500, and 800 MHz) to ensure a consistent comparison with the QDM measurements. Each antenna provides a different trade-off between penetration depth and spatial resolution. The 250 MHz antenna provides greater penetration depth but relatively lower spatial resolution. The 800 MHz antenna provides higher resolution for shallow structures but suffers from increased signal attenuation. The 500 MHz antenna represents an intermediate compromise. We acquired continuous GPR profiles along both survey Lines A and B using all three antenna frequencies. Based on the field-recorded survey procedure, the GPR was operated at approximately 1.0–1.1 m/s, corresponding to an estimated acquisition time of approximately 80–90 s for each 90 m survey pass. The principal section-level quantitative comparison with the QDM was based on the A-line profiles, whereas the B-line data were used as supporting observations for assessing the consistency of selected radar features.

The GPR data processing and analysis included time-zero correction, background removal, time-power gain (TPG), Hilbert-envelope calculation, and RMS-amplitude analysis. These procedures were applied independently to the 250, 500, and 800 MHz datasets.

Time-zero correction was performed to establish a consistent two-way travel-time reference within each frequency dataset. Background removal was then applied to suppress direct-wave energy, antenna ringing, and other signal components that persisted along the survey direction. A representative background trace was generated by averaging all traces over the entire 90 m survey line and was subsequently subtracted from each individual trace.

TPG was applied to improve the visibility of reflections at later two-way travel times. The gain coefficient was set to 2, 1, and 0.5 for the 250, 500, and 800 MHz datasets, respectively. The gain was progressively varied along the time axis to reduce the dominance of strong early-time responses and improve the relative visibility of weaker reflections occurring at later times. Because gain control modifies the original signal amplitudes, the gain-controlled profiles were interpreted in terms of relative reflection patterns rather than as calibrated measurements of absolute reflected energy.

For the envelope-based analysis, the amplitude envelope of each processed GPR trace was calculated as the magnitude of the analytic signal obtained using the Hilbert transform. The envelope RMS amplitude for each trace was then calculated as

(2) $$A_{RMS}=\sqrt{\frac{1}{N}\sum_{i=1}^{N}{e_{i}}^{2}}$$

where $e_{i}$ is the Hilbert-envelope amplitude at the *i*th time sample and *N* is the total number of samples included in the analyzed trace. The Hilbert-envelope and RMS-amplitude calculations were performed independently for the 250, 500, and 800 MHz datasets.

For the section-level analysis, the trace-level RMS envelope amplitudes were averaged over the spatial positions belonging to each pavement section. These section-level values were subsequently used for the quantitative comparison reported in Table 4 and Section 3.4.

The resulting RMS amplitudes can be affected by antenna response, bandwidth, signal attenuation, system sensitivity, and the frequency-specific gain settings. Therefore, the absolute RMS-amplitude values were not interpreted as directly comparable quantitative measurements across frequencies. Instead, the analysis focused on relative spatial variation among pavement sections within each frequency dataset and on the consistency of section-dependent patterns across the three frequencies. Processing for both sensing modalities was conducted offline, but computational time was not systematically benchmarked and is therefore not reported as a performance metric.

### 2.5. Repeated Measurements for Disturbance Characterization

Several disturbance sources influence outdoor magnetic field measurements, including sensor drift, temporal geomagnetic variation [20], transient magnetic interference, nearby moving vehicles, operator movement, and electronic noise. Rather than treating these disturbances as unavoidable sources of measurement uncertainty, we first characterized their temporal variability through repeated stationary measurements.

We selected representative spatial points in each pavement section and conducted nine repeated stationary measurements using the same measurement protocol and sensor configuration. Measurements were repeated as extensively as practicable within the available field-testing period and acquisition constraints, resulting in nine sessions at each of the 16 repeat locations (144 repeated acquisitions in total). Environmental conditions were not held constant across measurement sessions; ambient conditions and measurement times varied during the field campaign, consistent with the intended field-based evaluation. Each repeated measurement yielded 1001 magnetic field samples acquired over an effective recording period of approximately 37 s. We did not design these repeated measurements to estimate target detectability. Instead, we used them to quantify temporal stability, within-point variability, transient spike characteristics, baseline drift, and disturbance magnitude before conducting the dense spatial survey.

### 2.6. Disturbance-Informed Quality Control

Based on the repeated measurements, we designed a disturbance-informed preprocessing framework to improve the consistency of the dense spatial magnetic survey. The framework consisted of four sequential stages: (1) removal of the initial stabilization period; (2) identification and removal of transient disturbances; (3) correction of gradual baseline variation; and (4) estimation of a representative magnetic field value.

**Figure 5:**
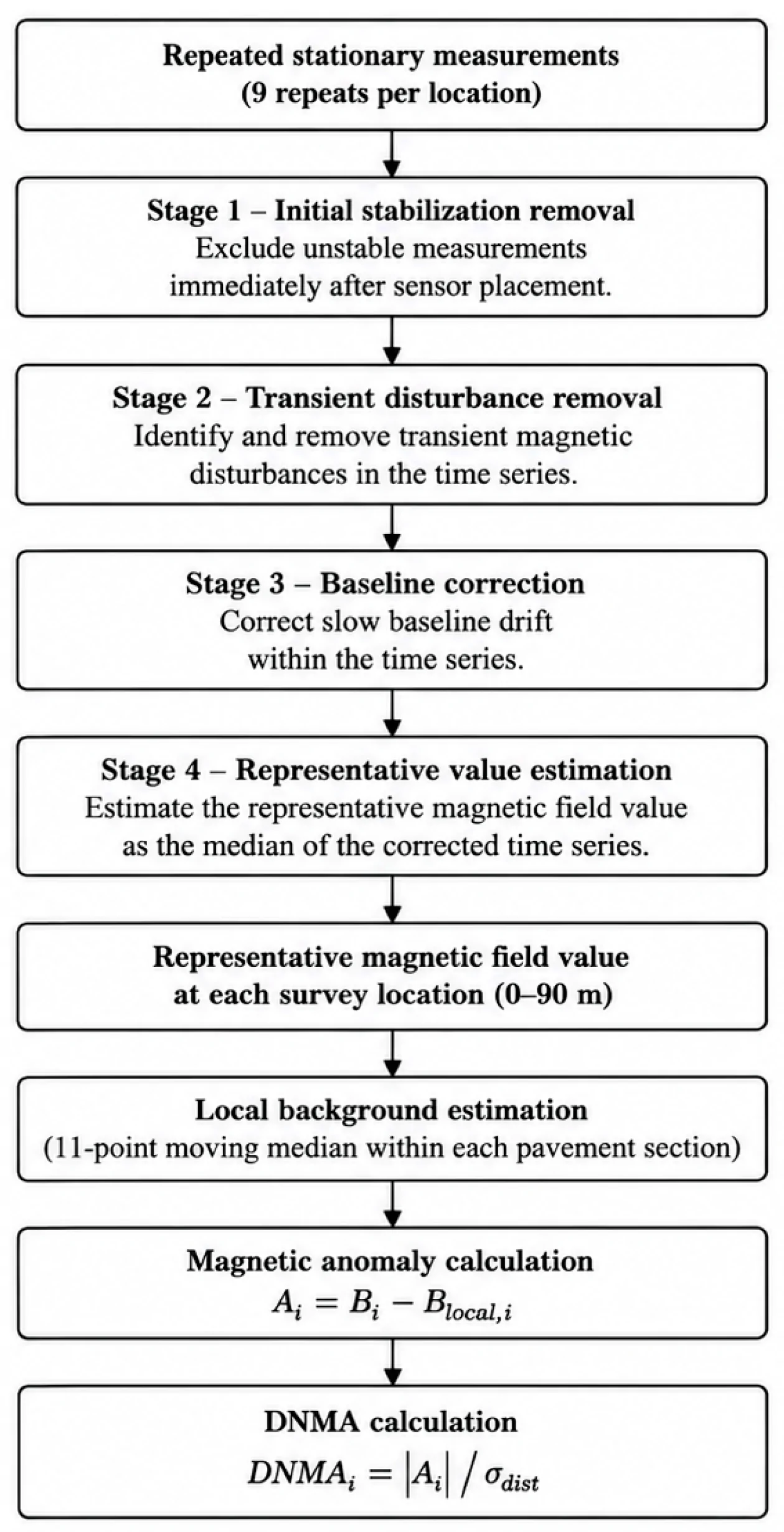
Disturbance-informed preprocessing framework.

We estimated the representative magnetic field at each survey spatial point using the median of the corrected time series. Compared with the arithmetic mean, the median is less sensitive to occasional disturbances and provides a more robust estimate of the stationary magnetic field. Figure 5 illustrates the disturbance-informed preprocessing framework.

### 2.7. Magnetic Anomaly Estimation

To identify localized magnetic disturbances potentially associated with buried ferromagnetic targets, we estimated a local background magnetic field at each 1 m survey spatial point using an 11-point moving median window (±5 m), providing a local background estimate while limiting the influence of localized magnetic excursions. The principal section-level pattern was preserved when 7- and 15-point windows were applied. We computed the moving median independently within each pavement section rather than across the entire 90 m survey line to avoid bias in the local background estimate near pavement-section boundaries, where background magnetic characteristics differed between pavement types.

The magnetic anomaly at survey spatial point *i* was calculated as

(3) $$A_{i}=B_{i}-B_{local,i}$$

where $B_{i}$ is the representative magnetic field at survey spatial point *i*, and $B_{local,i}$ is the local background magnetic field estimated from neighboring survey spatial points using the moving median filter.

Because the pavement sections were expected to exhibit different levels of background magnetic variability, applying a single fixed threshold to the raw magnetic anomaly across the entire survey line would not provide a consistent basis for comparison. A threshold suitable for sections with relatively low background variability could identify many spatial points in reinforcement-dense sections as anomalous, whereas a threshold calibrated for reinforcement-dense sections could overlook comparatively small but meaningful anomalies in quieter sections.

To facilitate consistent comparison among all pavement sections, we introduced the Disturbance-Normalized Magnetic Anomaly (DNMA),

(4) $$DNMA_{i}=\frac{|A_{i}|}{\sigma_{dist}}$$

where $\sigma_{dist}$ denotes the disturbance level estimated from the repeated stationary measurements. Specifically, $\sigma_{dist}$ was defined as the median within-point median absolute deviation (MAD = 6.7 nT) obtained from the nine-repeat disturbance characterization described in Section 2.5. We used a single spatial-point-independent disturbance estimate as the normalization reference for the entire survey line rather than a spatial-point-specific noise estimate. This estimate represents the typical temporal variability of the measurement system rather than the background characteristics of individual pavement sections. Consequently, DNMA expresses anomaly magnitude as a multiple of the typical measurement uncertainty instead of raw nanotesla values, facilitating comparison among pavement sections with substantially different background magnetic variability. Using this normalized anomaly score, we evaluated magnetic responses from different pavement sections relative to a common disturbance-based reference.

### 2.8. Comparison of Quantum Magnetometer and GPR

We compared quantum magnetometer measurements with GPR responses at nominally corresponding survey distances along the test section. At each survey spatial point, we compared magnetic anomalies with GPR reflection responses extracted from the 250, 500, and 800 MHz datasets. Rather than determining which sensing modality performed better, we focused on identifying spatial points detected by both sensors, spatial points primarily detected by the quantum magnetometer, spatial points primarily detected by GPR, and their complementary sensing characteristics.

In addition to the point-wise comparison, section-level QDM and GPR responses were quantitatively compared across the different pavement environments. The QDM analysis used the representative magnetic-field and anomaly metrics described above, whereas the GPR analysis used the envelope RMS amplitude calculated independently for each antenna frequency. Spatial correspondence between the two modalities was further evaluated using Spearman rank correlation, and post hoc ROC analysis was used to assess point-level discrimination against the documented target locations.

**Figure 6:**
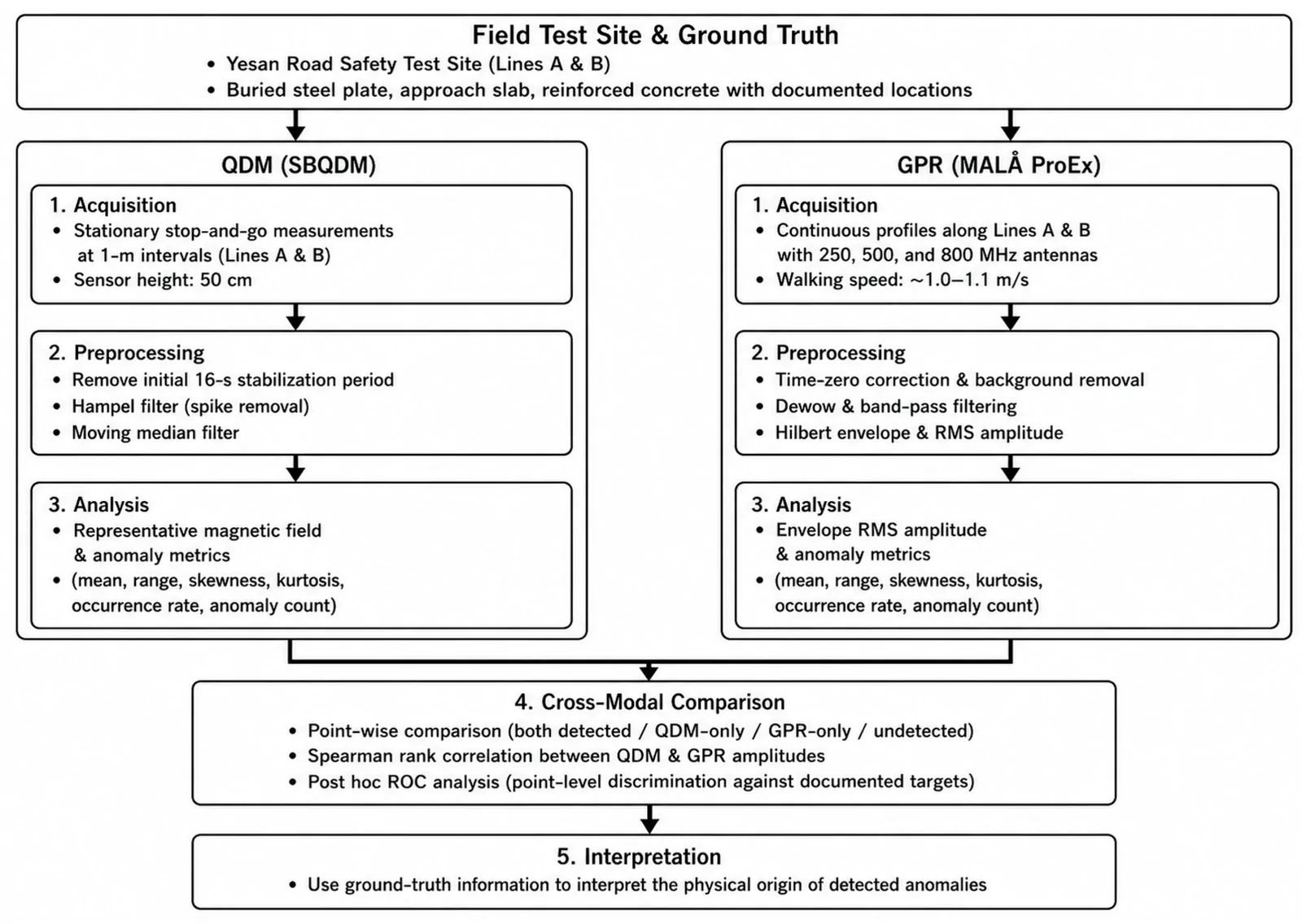
Schematic workflow of the complete methodology, showing parallel QDM and multi-frequency GPR acquisition, modality-specific processing and analysis, and subsequent cross-modal comparison and interpretation.

Figure 6 summarizes the complete methodological workflow, linking field acquisition and modality-specific preprocessing to QDM and GPR analysis, cross-modal comparison, and final interpretation. After completing the comparison, we used the ground-truth information only to interpret the physical origin of each detected anomaly.

### 2.9. Statistical Analysis

We analyzed the repeated magnetic measurements using robust statistical measures to minimize the influence of transient disturbances. For each measurement, we calculated the representative magnetic field as the median of the preprocessed time series acquired from the 1001 recorded samples. We then quantified within-point variability using the MAD [21] and between-session reproducibility using the standard deviation and coefficient of variation. We compared the spatial distribution of magnetic anomalies with GPR reflection responses using nominally corresponding survey distances. Because our objective was to evaluate complementary sensing characteristics rather than develop a target-classification model, we did not employ supervised learning or machine-learning algorithms. As a post hoc quantitative assessment of point-level detection performance, documented target locations were used as binary ground truth for receiver-operating-characteristic (ROC) analysis. The area under the ROC curve (AUC) was calculated for QDM DNMA and the 500 MHz GPR envelope RMS amplitude, with 95% confidence intervals. Because the ground-truth inventory was consulted after the original signal processing, this analysis was considered retrospective rather than a prospective blind-validation test. To evaluate section-dependent spatial variability, absolute point-to-point differences in representative QDM magnetic field were compared among the four pavement sections using the Kruskal–Wallis test, followed by Holm-adjusted Mann–Whitney U tests. GPR RMS envelope amplitudes were aggregated to 1-m spatial bins and analyzed separately for each antenna frequency using the same Kruskal–Wallis framework. Spearman correlation coefficients are reported with 95% confidence intervals estimated by paired bootstrap resampling of spatial points within each pavement section.

We performed all data processing and statistical analyses in Python 3.10 using NumPy, pandas, and SciPy. We used NumPy and pandas for data handling and preprocessing. We used SciPy for statistical analyses, including Spearman rank-correlation tests. We generated all figures using Matplotlib v.3.10.

## 3. Results

### 3.1. Repeatability of Quantum Magnetic Measurements

**Figure 7:**
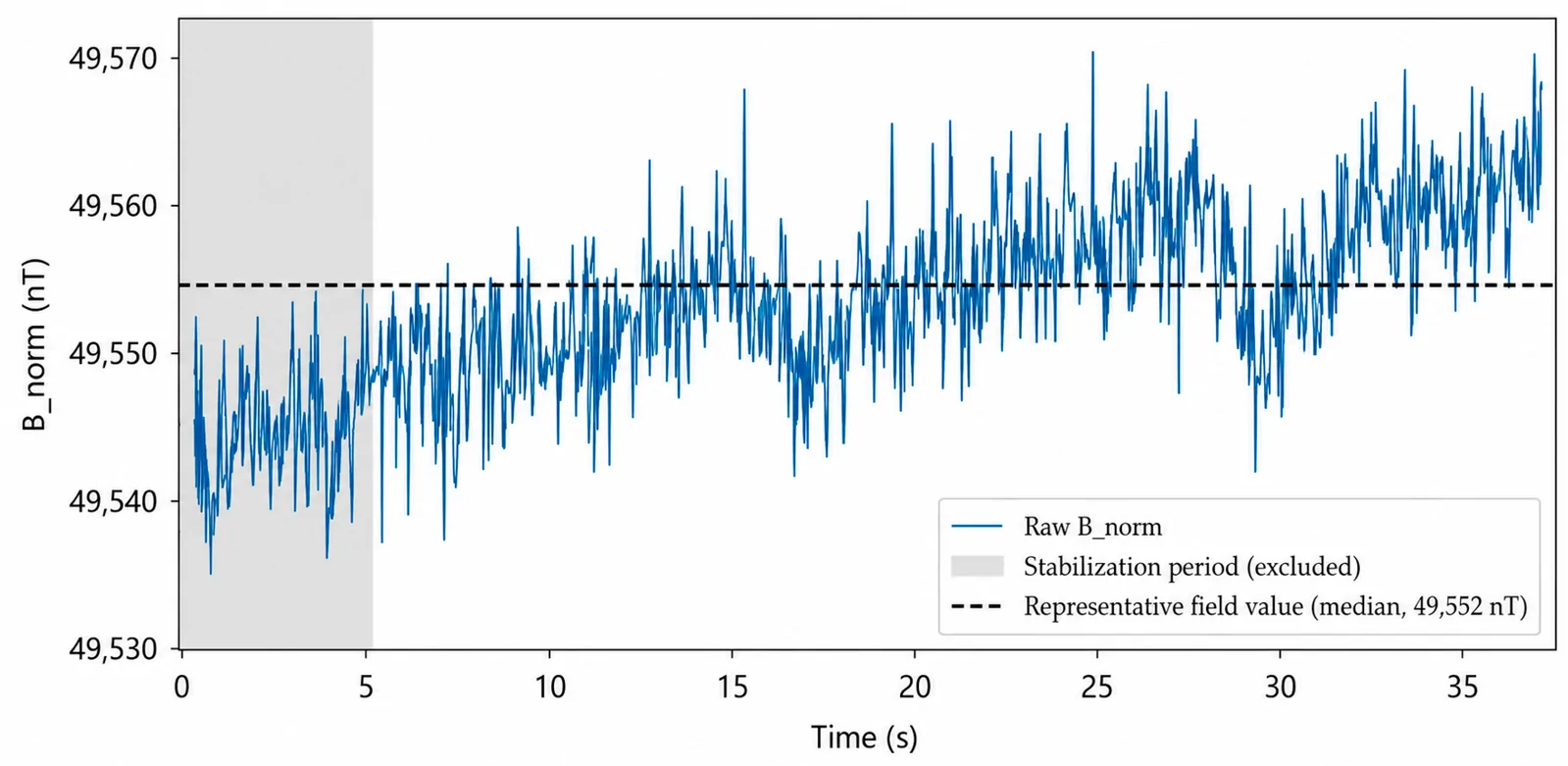
Representative repeated stationary magnetic measurements obtained using the same measurement protocol.

Repeated stationary measurements demonstrated high temporal stability of the QDM under field conditions across the ascon, approach slab, and reinforced concrete sections. Figure 7 illustrates representative time-series data obtained from the repeated measurements. Although small temporal fluctuations were observed, the magnetic field remained generally stable after the initial stabilization period, with no systematic drift or abrupt long-term instability throughout the repeated acquisitions.

For each session, we calculated the representative magnetic field as the median of the preprocessed time series and quantified within-session variability using the MAD. The median within-session MAD ranged from 4.5 to 7.8 nT across the sixteen spatial points, and 143 of 144 individual sessions remained below 10 nT (99.3%; 95% CI: 96.2–100.0). By contrast, the session-to-session standard deviation depended strongly on the pavement type: 409–477 nT in the ascon section (coefficient of variation, 0.83–0.95%), 548–1594 nT in the approach slab section (1.16–2.56%), and 714–4313 nT in the reinforced concrete section (1.08–7.80%).

**Figure 8:**
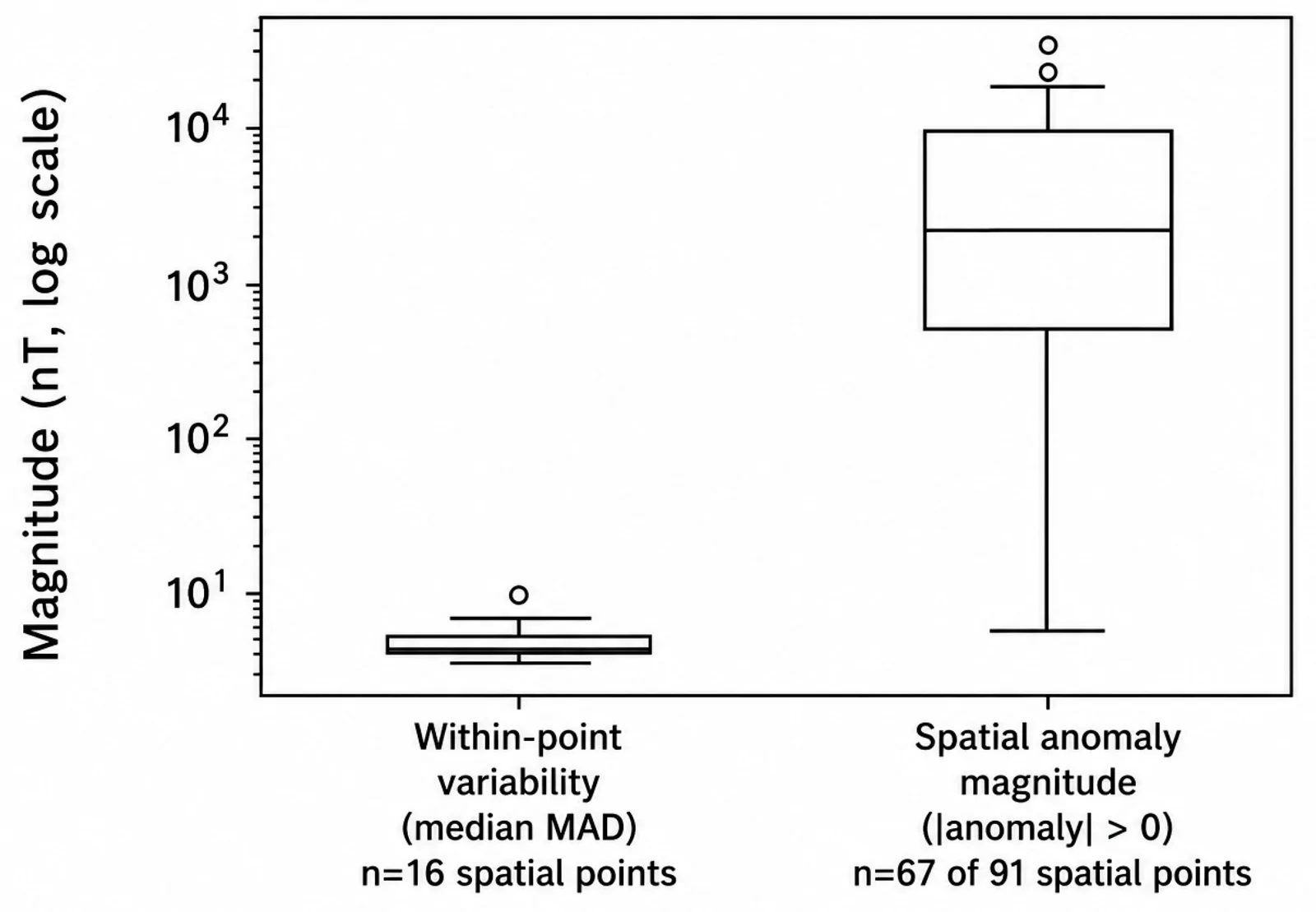
Comparison of within-point temporal variability (median MAD, *n* = 16 spatial points) and spatial magnetic anomaly magnitude, including only survey points with non-zero anomaly magnitudes (67 of 91 survey points); the y-axis is shown on a logarithmic scale.

Overall, temporal variability remained substantially smaller than the spatial magnetic variations observed along the survey line (Figure 8). While within-point magnetic variability remained within a relatively narrow range, magnetic anomalies associated with buried ferromagnetic structures reached magnitudes several times greater than the temporal fluctuations. These results indicate that the dominant magnetic anomalies identified during the survey primarily reflect subsurface conditions rather than random measurement noise.

The repeated measurements also revealed intermittent transient disturbances that appeared as short-duration magnetic spikes rather than persistent baseline shifts. Because these events were not repeatable across sessions at the same spatial point, we interpreted them as temporary environmental disturbances rather than signatures of buried targets.

Finally, the repeated measurements demonstrated the detection boundary addressed in RQ2. Along the ascon B-line, six adjacent spatial points (0–5 m) included one documented steel plate (0 m, 0.2–0.5 m depth), two loosened-ground zones (2–3 m), one EPS void (4 m), and one unreinforced concrete drain pipe (5 m). Only the steel-plate spatial point produced a distinct magnetic level: its session-mean $B_{norm}$ (49,307 nT) was separated from all five neighboring spatial points by approximately 850–1200 nT, roughly two to three times the between-session standard deviation (409–477 nT). In contrast, the loosened-ground zones, the EPS void, and the non-ferromagnetic drain pipe did not show similarly distinct magnetic separation from the target-free spatial point at 1 m, with session means clustered within 50, 150–50, 510 nT.

### 3.2. Spatial Magnetic Anomaly Survey (0–90 m)

**Figure 9:**
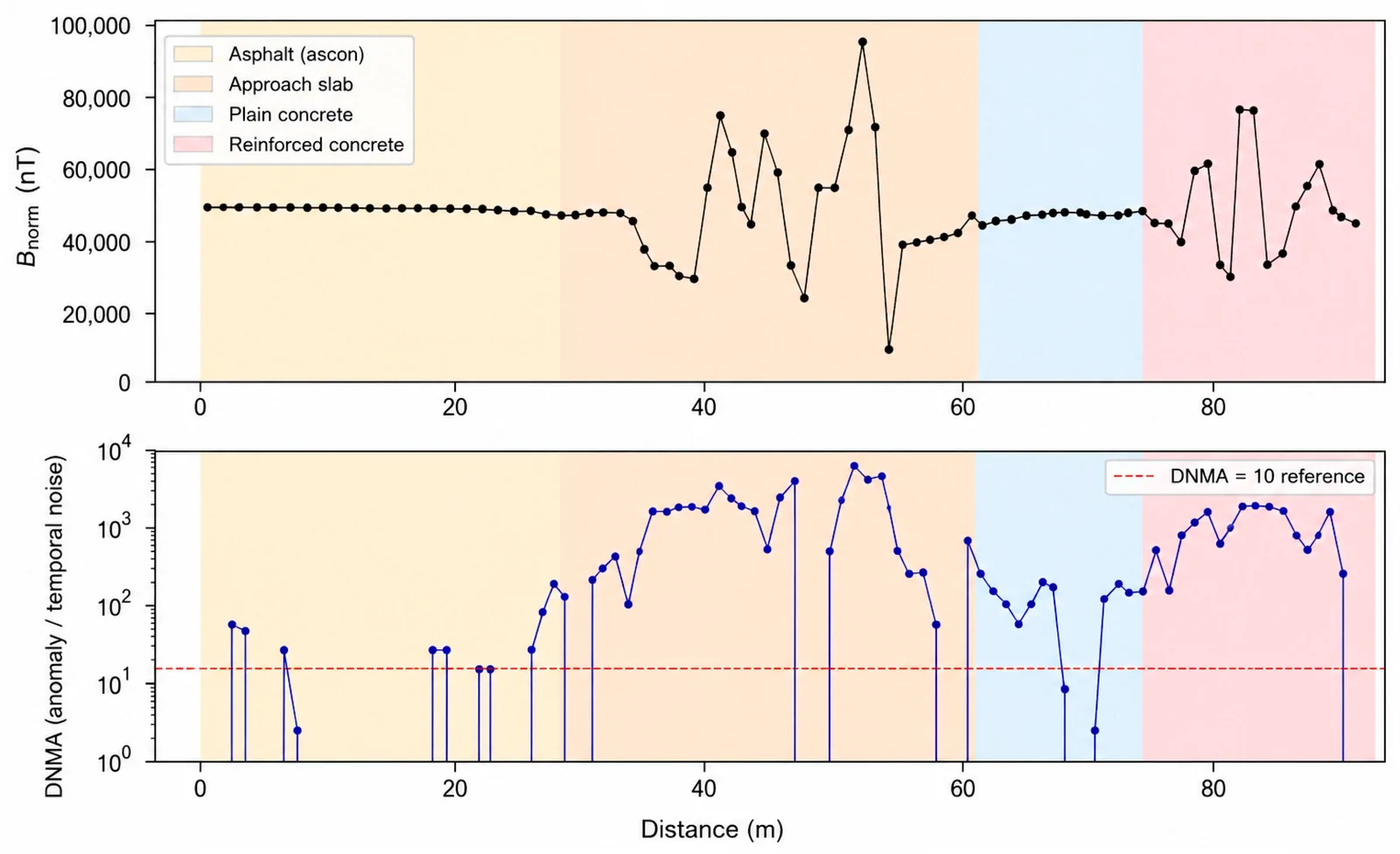
Representative magnetic field magnitude (**top**) and Disturbance-Normalized Magnetic Anomaly, DNMA (**bottom**), across the 0–90 m survey line. Shaded bands indicate the four pavement sections.

Following the repeatability characterization in Section 3.1, we conducted a dense magnetic survey at 1 m intervals along the entire 90 m test line, yielding 91 survey spatial points (0–90 m). Boundary spatial points at 30 m and 60 m were measured twice as part of the adjoining pavement sections, resulting in 93 individual acquisitions. Figure 9 summarizes the spatial distribution of the representative magnetic field and DNMA values across the survey line.

The spatial distribution of the representative magnetic field differed markedly among the four pavement sections (Table 4 and Figure 9). In the ascon section (0–29 m), B_norm varied smoothly over a range of 1972 nT (SD = 529 nT). The corresponding DNMA profile revealed a small number of discrete, localized peaks (near 2–3 m, 6 m, and 27–29 m, with smaller peaks near 20–24 m) that exceeded the surrounding background by more than one order of magnitude. Because the repeated measurements (Section 3.1) showed that the documented loosened-ground zones and non-ferromagnetic voids did not produce distinguishable magnetic signatures, these localized peaks are unlikely to originate directly from those documented features (Table 2). They may instead reflect unrecorded shallow ferromagnetic material, local background heterogeneity, or residual processing effects.

The anomaly cluster near 27–29 m lies within the ±5 m moving-background window of the pavement boundary at 30 m; therefore, part of the observed anomaly may result from the boundary-smoothing effect described in Section 2.7 rather than from a single discrete target.

The plain-concrete section (60–74 m), which contains no reinforcing steel, exhibited a similarly narrow magnetic range (2718 nT, SD = 749 nT) and a relatively quiet DNMA baseline interrupted by only a few moderate anomalies.

In contrast, the approach-slab (30–60 m) and reinforced-concrete (75–90 m) sections exhibited spatial variability that was one to two orders of magnitude greater than that of the two unreinforced sections, with B_norm ranges of 100,156 nT and 47,745 nT, respectively (Table 4). Inspection of the corresponding raw time-series data confirmed that these elevated magnetic levels remained stable throughout each measurement period. This observation suggests that the large magnetic variations primarily reflect the local magnetic environment rather than transient disturbances or sensor artifacts.

Because the point-to-point magnetic variation within these two sections was comparable to, or even greater than, the localized anomalies that the DNMA formulation (Section 2.7) was designed to isolate, the normalized anomaly score lost much of its discriminative capability. Consequently, nearly every spatial point in these sections exhibited DNMA values well above the reference disturbance level used elsewhere along the survey line. This behavior is consistent with the broad magnetic fluctuations observed in Figure 9, where the magnetic response appears to be dominated by distributed ferromagnetic components within the reinforced pavement structure rather than by isolated buried ferromagnetic targets. Post hoc ROC analysis showed weak point-level discrimination across the complete survey, with an AUC of 0.495 (95% CI: 0.370–0.609) for QDM DNMA and 0.472 (95% CI: 0.343–0.612) for the 500 MHz GPR envelope RMS amplitude. Thus, neither metric demonstrated reliable general target/non-target discrimination under the present field configuration. Point-to-point magnetic variation differed significantly among pavement sections (Kruskal–Wallis H(3) = 56.16, *p* < 0.001). Holm-adjusted pairwise comparisons showed that the approach-slab and reinforced-concrete sections did not differ significantly from one another (adjusted *p* = 0.656), whereas both exhibited significantly greater spatial variation than the two less magnetically heterogeneous sections (all adjusted *p* < 0.001).

### 3.3. Multi-Frequency GPR Results (0–90 m)

**Figure 10:**
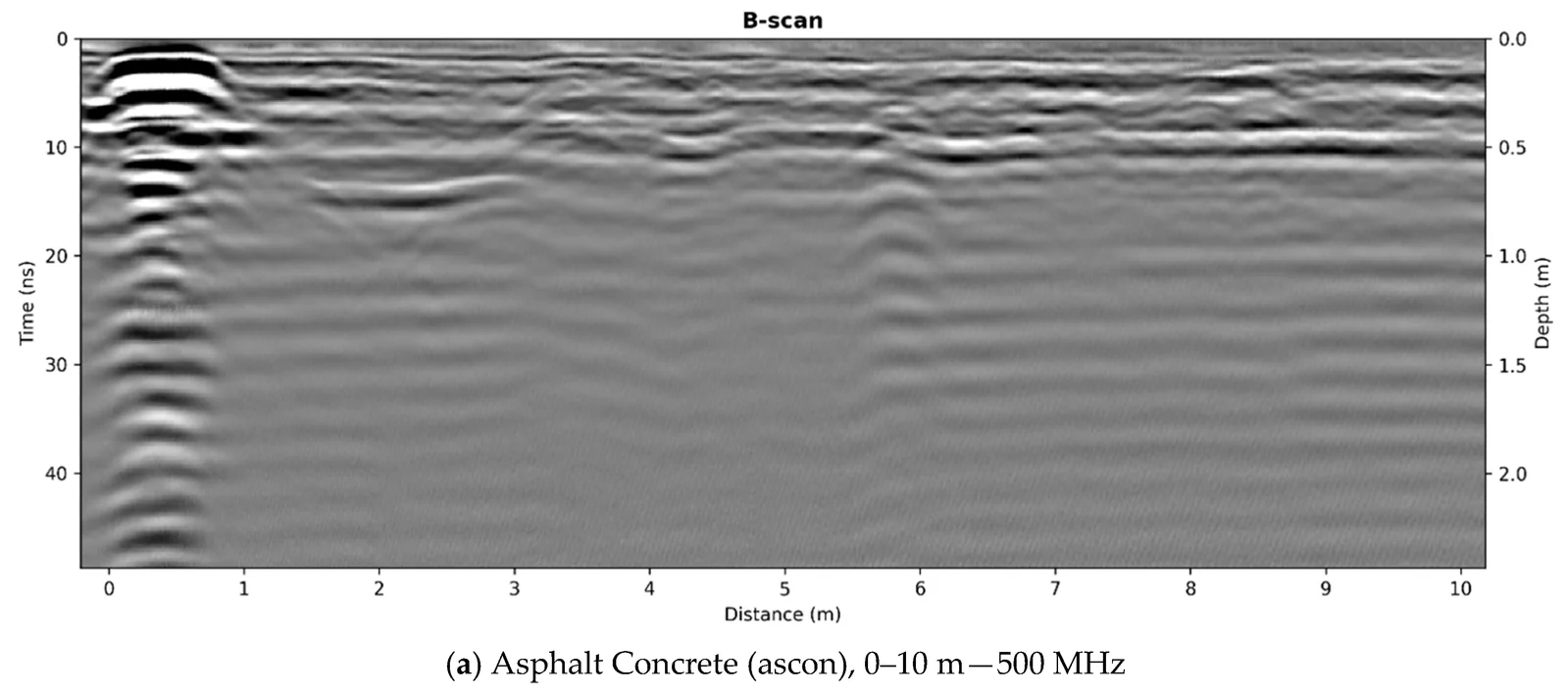

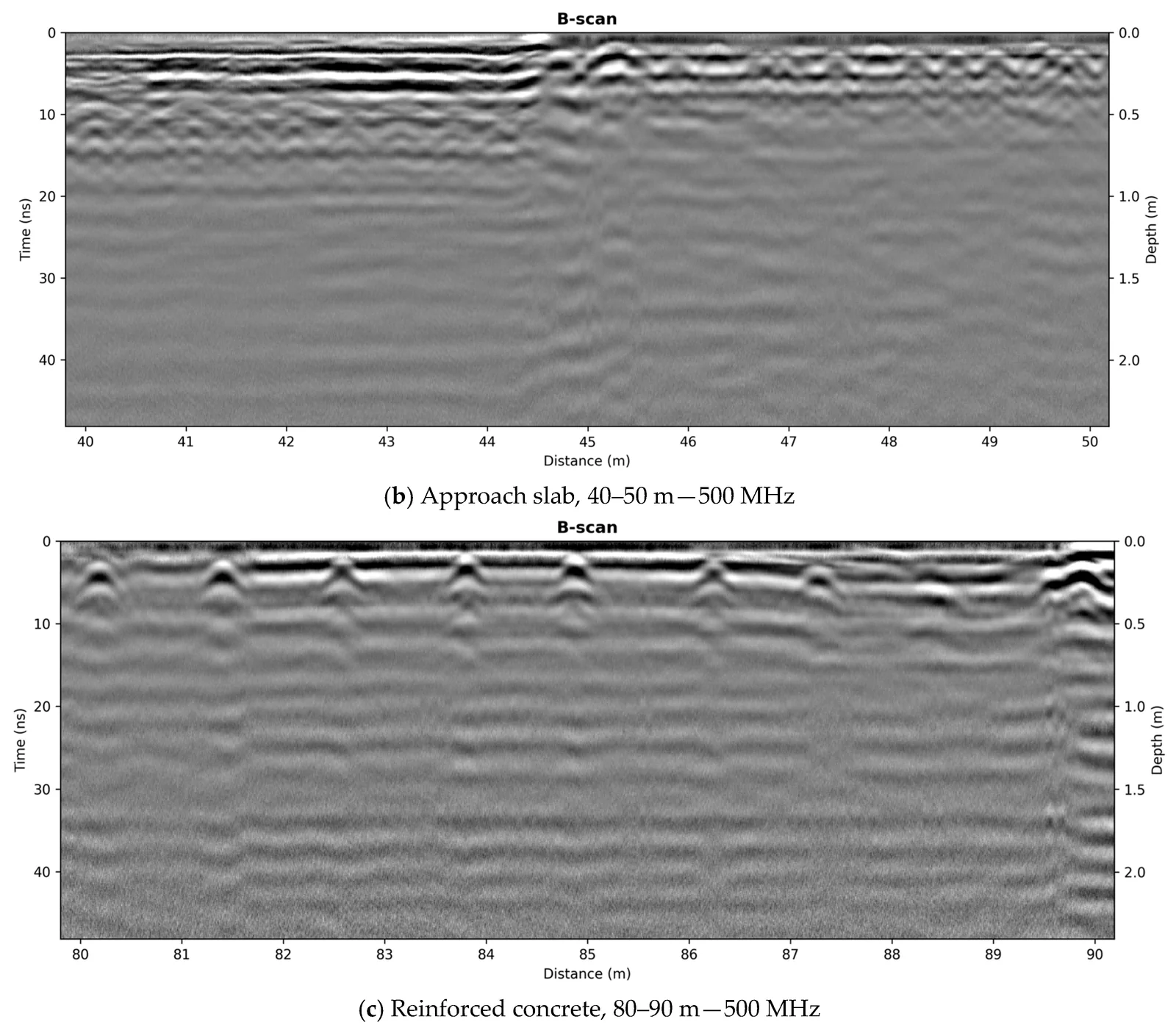
Representative 500 MHz GPR B-scans illustrating three characteristic subsurface conditions encountered at the test site: (**a**) asphalt concrete with a shallow buried steel plate producing a distinct hyperbolic reflection; (**b**) the approach slab section showing the transition near 44–46 m; (**c**) the reinforced-concrete section characterized by regularly spaced shallow reflections.

Despite spatial variation in the GPR envelope RMS amplitude across the survey line, the radargrams retained interpretable shallow subsurface features. In particular, the reinforced-concrete section (75–90 m) displayed a series of clear, regularly spaced hyperbolic reflectors at approximately 1.0–1.3 m intervals in the shallow subsurface (Figure 10, bottom panel). Given their relatively wide spacing, these periodic features were not attributed specifically to individual reinforcing bars. Their precise structural origin could not be conclusively determined from the available GPR data and site documentation. No comparable periodic reflector pattern was observed in the ascon or plain concrete sections. Figure 10 presents representative 500 MHz GPR B-scans illustrating characteristic subsurface responses across the test site. The ascon section (Figure 10a) shows a distinct hyperbolic reflection produced by the shallow buried steel plate, providing a reference example of a clear target response under relatively uncomplicated subsurface conditions. The approach slab section (Figure 10b) illustrates the transition from 44 to 46 m, while the reinforced-concrete section (Figure 10c) exhibits regularly spaced shallow reflections of uncertain structural origin.

### 3.4. Comparison of Quantum Magnetometer and GPR (Complementary Sensing)

**Figure 11:**
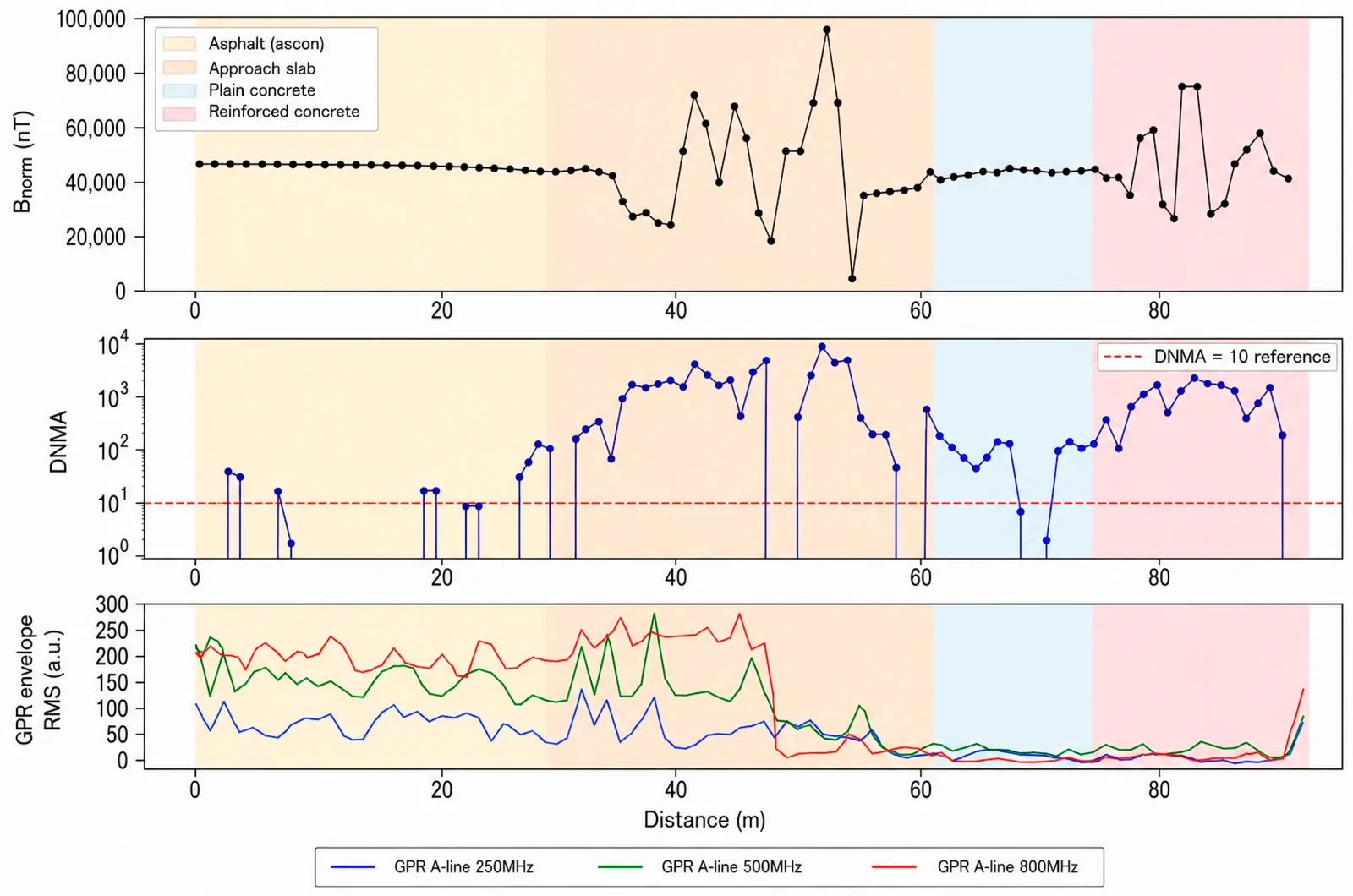
Comparison of quantum magnetometer profile (B_norm, (**top**); DNMA, middle, log scale) with the multi-frequency GPR envelope RMS amplitude profiles (A-line, (**bottom**)) across the 0–90 m survey line. Shaded bands indicate the four pavement sections. The figure highlights the contrasting responses of the two sensing modalities across different pavement structures.

Figure 11 compares the representative magnetic field, DNMA values, and multi-frequency GPR envelope RMS amplitude along the shared 0–90 m survey coordinate (Section 2.8). For the section-level comparison, the trace-level RMS envelope amplitudes defined in Section 2.4 were averaged within each pavement section, with the resulting values reported in Table 4. Because the three antenna frequencies have different system responses and gain coefficients, their absolute RMS amplitudes were not interpreted as directly comparable across frequencies; comparisons were made among pavement sections within each frequency. At the section level, the two sensing modalities showed contrasting responses: the sections exhibiting elevated and highly variable magnetic responses (approach slab and reinforced concrete) corresponded to those where the GPR envelope RMS amplitude was substantially reduced (Figure 11, Table 4). At the finer, point-to-point scale, however, the correspondence was weaker. Spearman correlations between the absolute magnetic anomaly and the per-frequency GPR envelope RMS amplitude, computed within each pavement section, ranged from −0.65 (95% CI: −0.89 to −0.19) to +0.38 (95% CI: 0.02 to 0.68) and were inconsistent in magnitude and sign across antenna lines, frequencies, and pavement sections; 21 of the 24 bootstrap confidence intervals included zero. Thus, the quantitative complementarity observed here reflects two distinct section-level indicators—magnetic heterogeneity and radar-response amplitude—rather than improved point-level target-detection accuracy. Section-level differences in the 1-m-aggregated GPR RMS envelope amplitude were significant at all three frequencies (Kruskal–Wallis tests: 250 MHz, H = 60.57, *p* < 0.001; 500 MHz, H = 57.87, *p* < 0.001; and 800 MHz, H = 55.63, *p* < 0.001).

### 3.5. Cross-Session Validation of the Repeat-Based Baseline

The repeat-based baseline and the dense spatial survey were acquired in temporally separated measurement sessions, allowing a cross-session evaluation of RQ3, namely whether a site-specific baseline established through repeated measurements was consistent with subsequent single-pass measurements. The repeat-based baseline was derived from nine measurement sessions conducted over seven field days between 8 May and 12 July 2026. The corresponding dense spatial measurements were subsequently acquired on 14 July 2026 for the approach-slab section and on 15 July 2026 for the reinforced-concrete section. Thus, the dense spatial measurements used for this comparison were obtained approximately two to three days after the final repeated-measurement session.

At the ten A-line spatial points where the two datasets coincided (41–45 m in the approach-slab section and 86–90 m in the reinforced-concrete section), all ten single-pass measurements fell within ±1.5 SD of their corresponding repeat-based baselines (10/10; 95% CI for the proportion: 69.2–100%). In particular, at the confirmed ferromagnetic target at 44 m, a cavity containing a steel plate at its base (Table 2), the single-pass survey value (47,073 nT) agreed with the repeat-based baseline (mean = 47,108 nT, 95% CI: 46,692–47,525 nT; SD = 542 nT) to within −0.06 SD. This agreement was particularly evident in the reinforced-concrete section, where the between-session standard deviation varied nearly six-fold between adjacent spatial points (714–4313 nT at 86–90 m). Despite this substantial variation in local baseline variability, all single-pass measurements remained within ±1.5 SD of their corresponding repeat-based baselines. A distinct single-pass magnetic response was also observed at the documented cavity-plus-steel-plate target at 46 m; however, this location was not included in the repeat-based cross-session comparison.

## 4. Discussion

### 4.1. Physical Basis of the Observed Complementarity

The section-level comparison presented in Figure 11 indicates that the quantum magnetometer and multi-frequency GPR provide complementary rather than redundant information for pavement characterization. This complementary behavior is reflected in the pattern observed in Section 3.4, where substantially reduced GPR reflectivity coincided with elevated and highly variable magnetic responses in the reinforcement-dense sections. This contrast is consistent with the distinct physical mechanisms underlying the two sensing modalities. GPR contrast arises from dielectric discontinuities along the propagation path, and embedded metallic components in reinforced structures can scatter and attenuate the radar wavefront, producing clutter that degrades radargram interpretability [22,23,24].

In contrast, the QDM responds to magnetic fields associated with ferromagnetic materials within its sensing environment. Magnetic anomaly amplitude depends strongly on sensor-to-source distance as well as the amount, geometry, orientation, and magnetic properties of the ferromagnetic material. The substantially greater magnetic variability observed in the approach-slab and reinforced-concrete sections is therefore consistent with the presence of distributed ferromagnetic components within these structures. This is reflected in the 100,156 nT and 47,745 nT magnetic ranges observed in the approach-slab and reinforced-concrete sections, respectively (Section 3.2), compared with the sub-3000 nT ranges measured in the two unreinforced sections. Unlike GPR, the NV-diamond magnetometer does not rely on dielectric contrast for target detection and is therefore not affected by dielectric scattering in the same manner. This physical characteristic helps explain why the quantum magnetometer remained informative in sections where GPR reflectivity was substantially reduced.

A potentially important source of transient magnetic interference was traffic on the nearby roadway, located approximately 50 m from the survey area. Passing vehicles contain substantial ferromagnetic components and may therefore have contributed to short-duration magnetic disturbances and cross-session variability, although their individual contributions could not be isolated quantitatively in the present field experiment.

This pattern parallels previous reports showing that combined magnetic and GPR surveys can recover buried features visible in only one of the two datasets [25]. It is also consistent with sensor-fusion approaches developed for buried-ordnance and utility detection, where GPR, electromagnetic-induction, and magnetometer data reduce false alarms when used together rather than individually [18]. Similar concepts have been demonstrated in airborne GPR–magnetometer systems for landmine detection [19] and in autonomous multi-sensor robotic platforms that integrate complementary nondestructive evaluation techniques for civil infrastructure inspection [26].

The cross-session comparison results presented in Section 3.5 further suggest that absolute magnetic field magnitude alone may not be sufficient for interpreting localized anomalies in magnetically heterogeneous pavement environments. Instead, expressing single-pass observations relative to a locally established repeat-based baseline provided a consistent statistical framework despite substantial differences in local background variability. This finding complements the physical interpretation presented above by demonstrating that repeat-based normalization can compensate for spatial-point-dependent magnetic variability while preserving meaningful anomaly information.

The present study applies this complementary sensing concept to a portable NV-diamond magnetometer in a pavement environment. The magnetic responses observed at the documented steel-plate targets support its potential to provide complementary information to GPR when ferromagnetic materials are present. However, because no conventional magnetometer was operated in parallel over the same targets, the present results do not establish superior sensitivity, accuracy, or dynamic range of the QDM relative to established magnetometer technologies. Such performance differences would require a controlled side-by-side comparison using the same measurement protocol. Accordingly, the practical value demonstrated here lies in obtaining magnetic-contrast information that is physically distinct from the dielectric response measured by GPR, rather than in a demonstrated performance advantage over conventional magnetometers. Conversely, the absence of consistent localized responses to the evaluated non-ferromagnetic conditions, the observed environmental magnetic variability, and weak general point-level discrimination represent practical limitations of the QDM in the present inspection configuration. The steel-plate response observed near the 0 m location further supports this interpretation by demonstrating that the QDM consistently responded to shallow ferromagnetic targets under substantially different pavement conditions. Importantly, this complementary behavior was not supported by a single target alone. Additional ferromagnetic targets at the shallow 0 m location and the neighboring 46 m cavity-plus-steel-plate location also produced magnetic responses consistent with the observations at the 44 m target, indicating that the proposed interpretation is reproducible across multiple target configurations.

### 4.2. Implications for Infrastructure Inspection Practice

The complementary sensing behavior discussed in Section 4.1 has important implications for practical infrastructure inspection. From a practical inspection standpoint, the two sections where GPR reflectivity was degraded (approach slab and reinforced concrete) are exactly those where reinforced-concrete bridge transitions are most prone to hidden defects and where conventional GPR interpretation is complicated by multiple reflections from reinforcement [22,23,24], a limitation that has motivated dedicated magnetic and electromagnetic reinforcement-characterization methods such as cover meters and GPR–EMI dual sensors [27,28,29]. The present results suggest that adding a quantum magnetometer alongside a routine multi-frequency GPR survey could provide complementary information in precisely these problematic sections, because both instruments can be operated along the same survey alignment in sequential surveys. This is consistent with the complementary, rather than competitive, objective stated in Section 1: neither sensor is presented here as a replacement for the other, and the greatest practical benefit appears where their individual limitations do not overlap.

However, although GPR supports quantitative analyses such as reflection-amplitude measurement, travel-time-based depth estimation, and dielectric-property estimation, these analyses were beyond the scope of the present study. Instead, standard radargram interpretation was adopted because the primary objective was to evaluate the complementary field performance of the QDM rather than to optimize GPR signal-processing techniques.

Importantly, the present results do not suggest that magnetic sensing is immune to reinforcement effects. While GPR exhibited reduced interpretability because of signal attenuation and multiple reflections, the QDM also showed diminished discrimination within reinforcement-dense sections, as reflected by the section-wide elevation of DNMA values. Rather than indicating that one sensor simply overcomes the limitations of the other, these findings demonstrate that the two modalities respond differently to the same structural environment and therefore provide complementary information for interpretation.

Although reinforcement reduced the discriminative performance of the QDM, the resulting section-wide elevation of DNMA may itself provide useful diagnostic information. A section-wide elevation of DNMA above the reference threshold, rather than a small number of discrete peaks, may indicate that the magnetic environment is dominated by distributed ferromagnetic components within the pavement structure and that interpretation based solely on GPR should therefore be undertaken with additional caution.

Because repeat-based normalization compensates for spatial-point-dependent magnetic variability, the cross-session comparison presented in Section 3.5 also suggests a practical framework for long-term structural monitoring. During an initial characterization survey, repeated stationary acquisitions (≈37 s each) establish a per-point baseline (mean and between-session SD) for the section of interest. Subsequent periodic inspections then require only a single-pass survey, with each reading evaluated as a deviation from its corresponding baseline. In the present study, the confirmed ferromagnetic target spatial point re-measured in a separate session agreed with its repeat-based baseline within 0.1 SD, and all ten co-located points remained within ±1.5 SD. Future measurements that fall outside the expected variability of the repeat-based baseline would warrant further investigation because they are more likely to reflect genuine subsurface change than ordinary measurement variability. This repeat-then-monitor protocol helps address the inspection-versus-monitoring gap identified for quantum magnetometers in infrastructure applications [13].

### 4.3. Limitations

Several limitations should be considered when interpreting these results. First, we consulted the ground-truth inventory of buried-target coordinates, depths, and materials (Table 2) only after data processing and anomaly detection (Section 2.2), so the point-by-point correspondence between individual anomalies and specific targets described in Section 3 reflects a post hoc comparison rather than a blind detection test. Accordingly, the post hoc ROC analysis reported in Section 3.2 should be interpreted as a retrospective performance assessment rather than a prospective blind detection test.

Second, we acquired the GPR survey along two antenna lines (A and B; Section 2.1), and the present comparison relied primarily on the A-line. Establishing precise spatial registration between the GPR survey lines and the magnetometer survey path, and confirming that both surveys share a common distance origin, will be necessary before the weak point-to-point correlations reported in Section 3.4 can be interpreted quantitatively.

Third, the abrupt raw-signal-level transition observed at approximately 44–46 m in all three GPR frequencies and both antenna lines (Section 3.3, Figure 10) coincides with a confirmed ground-truth target at the 44 m survey point—a cavity containing a steel plate at a depth of 1.0–1.5 m (Table 2). This coincidence suggests that the transition is more likely to represent a genuine target-related change in subsurface reflectivity than an acquisition artifact. Although this interpretation is supported by an additional target response near the 0 m location in the B-line survey, the present evidence remains limited to a small number of coincident observations. Further confirmation through excavation or independent repeat surveys would strengthen this conclusion.

Finally, we conducted the present validation at a single, purpose-built test site. While this design enabled sensor comparison under controlled and well-documented conditions, replication at operational, non-test sites will be necessary to evaluate the generalizability of the section-level complementarity pattern reported here. Future studies should also examine a wider range of bridge-transition geometries, reinforcement configurations, and naturally deteriorated infrastructure to determine whether the observed complementarity remains robust under more diverse field conditions. Because environmental conditions and measurement times were not held constant across sessions, the observed cross-session variability may include both site-specific magnetic variation and uncontrolled environmental effects. The nine-session dataset should therefore be interpreted as a practical characterization of variability during the present field campaign rather than as establishing long-term baseline stability or a generally sufficient number of repetitions for QDM field assessment.

### 4.4. Future Work

Building on the complementary sensing concept and the disturbance-informed preprocessing framework validated in the present study (Section 2.6, Section 3.1 and Section 3.5), a logical next step is to develop a supervised or semi-supervised classification framework. The preprocessing framework presented here already provides robust baseline normalization; future work should extend it with data-driven classification rather than replacing it. Such a framework would combine magnetic features (e.g., DNMA and local gradient) with GPR features (e.g., envelope RMS amplitude and hyperbola presence), using the ground-truth inventory (Table 2) for training and validation. This approach would allow the section-level complementarity demonstrated here to be evaluated quantitatively at the level of individual buried targets.

Confirming the GPR line-to-path registration discussed above would also enable a fully quantitative, point-by-point sensor-fusion analysis instead of the section-level comparison presented in this study.

Future studies should additionally investigate statistical change-detection methods based on repeat-derived baselines so that long-term infrastructure monitoring can be automated. They should also investigate the long-term stability of repeat-based baselines under seasonal environmental changes and repeated operational inspections. Such validation will be essential before this repeat-based monitoring framework can be recommended for routine long-term infrastructure monitoring.

## 5. Conclusions

This study presented a field validation of complementary subsurface sensing using a QDM and multi-frequency (250, 500, and 800 MHz) GPR at a full-scale, purpose-built test site comprising ascon, approach-slab, plain-concrete, and reinforced-concrete pavement sections with a documented ground-truth inventory of buried targets (Table 2). All magnetic data were processed using a disturbance-informed preprocessing framework calibrated from repeated stationary measurements, and the four research questions posed in Section 1 can now be addressed directly based on the present findings.

Regarding field reproducibility (RQ1), the QDM achieved a within-session median MAD of 4.5–7.8 nT at all sixteen repeat spatial points, while session-to-session variability depended strongly on pavement type, ranging from 0.83–0.95% (coefficient of variation, CV) in the ascon section to as much as 7.80% in the reinforced-concrete section. These results show that within-session variability was substantially smaller than between-session variability, which depended strongly on pavement type and may include both site-specific magnetic variation and uncontrolled environmental effects.

Regarding the detection boundary (RQ2), detection was contingent on ferromagnetic composition. The documented shallow steel-plate target produced a magnetic level clearly separated from neighboring non-ferromagnetic locations, whereas the evaluated non-ferromagnetic subsurface features, including EPS voids and an unreinforced concrete drain pipe, did not produce similarly distinct magnetic separation from target-free ground. Additional responses at the cavity-plus-steel-plate locations were consistent with this composition-dependent pattern.

Regarding baseline characterization and validation (RQ3), a repeat-established baseline was compared with a subsequent single-pass survey acquired in separate measurement sessions: all ten co-located points agreed within ±1.5 between-session standard deviations, including agreement within 0.1 SD at the confirmed ferromagnetic target at 44 m (Section 3.5). Because the between-session standard deviation itself varied nearly six-fold between adjacent reinforced-concrete spatial points, single-pass magnitude alone may not be sufficient to distinguish genuine anomalies from reinforcement-related magnetic variability, whereas the per-point baseline provides a common statistical reference, supporting the repeat-then-monitor protocol proposed in Section 4.2.

Regarding complementarity with GPR (RQ4), the two sensing modalities showed complementary section-level responses. The quantum magnetometer remained informative where GPR envelope RMS amplitude was substantially reduced in reinforcement-dense sections, including the pronounced transition observed near 44–46 m, where the 800 MHz response decreased by approximately three- to five-fold, whereas GPR retained diagnostic value by clearly resolving discrete targets in the less complex pavement sections and structural features such as the periodic near-surface reflectors in the reinforced-concrete section.

Taken together, these findings demonstrate that the principal value of the QDM lies not in replacing GPR, but in providing complementary magnetic information in reinforcement-dense environments where radar performance is inherently limited. The present results therefore position the QDM as a complementary sensing channel that is particularly valuable in sections where GPR interpretation is challenged by dense structural reinforcement, while also providing one of the first field-based quantifications of repeatability, addressing an important validation gap for quantum magnetometers in civil infrastructure applications.

Although the present study demonstrated section-level complementarity under the tested field conditions, confirming this relationship at the level of individual buried targets—through excavation-confirmed ground truth, verified GPR line registration, and replication at operational sites—will be the focus of the next phase of this research program.

## Figures and Tables

**Table 1:** Comparison of selected magnetometer technologies and their relevance to complementary subsurface sensing with GPR.

Magnetometer	Principal Strengths	Principal Limitations	Relevance to GPR Complementarity
Fluxgate	Compact; vector sensing; mature field use	Offset/drift; moderate sensitivity	Detection/mapping of ferromagnetic objects
Proton-precession	Scalar absolute-field measurement; good stability	Lower sampling rate	Robust total-field magnetic surveying
Optically pumped	High sensitivity	Operating-range/environmental constraints depend on design	Detection of weak magnetic anomalies
NV-diamond QDM (this study)	Solid-state quantum sensing; operation in ambient geomagnetic field; vector-derived total-field measurement	Environmental/thermal variability; current field protocol relatively slow; field maturity lower	Provides magnetic contrast independent of GPR dielectric response

**Table 2:** Ground-truth information of buried targets.

Section	Distance (m)	Target Type	Top Depth (m)	Bottom Depth (m)	Note
Asphalt concrete	0	Steel plate	0.2	0.5	-
Asphalt concrete	2	EPS (void)	0.2	0.5	-
Asphalt concrete	5	EPS (void)	0.8	1.0	Adjacent to drain pipe
Asphalt concrete	11	EPS (void)	1.0	1.2	-
Asphalt concrete	14	EPS (void)	2.0	2.2	-
Asphalt concrete	17	EPS (void)	3.0	3.2	-
Asphalt concrete	20	EPS (void)	2.7	3.0	-
Asphalt concrete	23	EPS (void)	2.0	2.2	-
Asphalt concrete	26	EPS (void)	1.5	1.7	-
Asphalt concrete	29	EPS (void)	0.3	0.6	-
Approach slab	31	EPS (void)	0.7	1.0	-
Approach slab	33	EPS (void)	1.3	1.5	-
Approach slab	35	EPS (void)	0.7	1.0	-
Approach slab	38	EPS (void)	1.3	1.5	-
Approach slab	41	EPS (void)	1.5	1.7	-
Approach slab	44	Cavity + Steel plate	1.0	1.5	Plate at cavity base
Approach slab	46	Cavity + Steel plate	0.5	1.0	Plate at cavity base
Approach slab	49	EPS (void)	1.7	2.0	-
Approach slab	51	EPS (void)	1.2	1.5	-
Approach slab	54	EPS (void)	1.7	2.0	-
Approach slab	57	EPS (void)	0.7	1.0	-
Approach slab	59	EPS (void)	1.3	1.5	-
Plain concrete	61	EPS (void)	2.7	3.0	-
Plain concrete	62	EPS (void)	0.7	1.0	-
Plain concrete	65	Drain pipe	1.5	2.2	Unreinforced concrete pipe
Plain concrete	68	EPS (void)	1.2	1.5	-
Plain concrete	71	EPS (void)	1.7	2.0	-
Plain concrete	74	EPS (void)	2.2	2.5	-
Reinforced concrete	76	EPS (void)	2.2	2.5	-
Reinforced concrete	79	EPS (void)	1.7	2.0	-
Reinforced concrete	82	EPS (void)	1.2	1.5	-
Reinforced concrete	85	Drain pipe	1.5	2.2	Unreinforced concrete pipe
Reinforced concrete	88	EPS (void)	0.7	1.0	-
Reinforced concrete	89	EPS (void)	2.7	3.0	-

**Table 3:** Specifications of the quantum diamond magnetometer.

Characteristic	Specification
Manufacturer/Model	SBQuantum SBQDM
Sensing element	Nitrogen-vacancy (NV) center ensemble in synthetic diamond
Output	Vector magnetic field (four crystallographic axes) plus total field magnitude (*B*_norm_)
Effective sampling rate	Approx. 26.8 Hz (1001 samples over approximately 37 s)
Power requirement	24 V DC, 3 W
Operating temperature (calibrated)	5 °C to 35 °C
Sensitivity	<1 nT/√Hz at 1 Hz (estimated)
Measurement range	±200 µT
Measurement accuracy	~1 nT (estimated)
Signal-to-noise ratio (SNR)	Not specified as a fixed instrument-level value *

* SNR depends on the target-response magnitude and environmental magnetic noise.

**Table 4:** Zone-wise summary statistics of the QDM and GPR envelope RMS amplitude profiles across the 90 m survey line.

Zone	Distance Range (m)	*n*	B_norm Range (nT)	B_norm SD (nT)	GPR Envelope RMS Amplitude, 250 MHz (a.u.)	GPR Envelope RMS Amplitude, 500 MHz (a.u.)	GPR Envelope RMS Amplitude, 800 MHz (a.u.)
Ascon	0–29	30	1972	529	85.5	139.0	171.3
Approach slab	30–60	31	100,156	18,561	67.6	99.6	118.8
Plain concrete	61–74	14	2718	749	38.9	47.2	29.5
Reinforced concrete	75–90	16	47,745	15,117	37.0	52.7	41.5

## Data Availability

The datasets generated and analyzed during the current study are publicly available through Zenodo at https://doi.org/10.5281/zenodo.21441974. A description of the repository contents and file-naming conventions is provided in Appendix A.

## Abbreviations

CV	Coefficient of Variation
DNMA	Disturbance-Normalized Magnetic Anomaly
GPR	Ground-Penetrating Radar
MAD	Median Absolute Deviation
NV	Nitrogen Vacancy (center)
QDM	Quantum Diamond Magnetometer
RMS	Root Mean Square
SNR	Signal-to-Noise Ratio
TPG	Time Power Gain

## Appendix A. Description of the Repository Contents and File-Naming Conventions

### Appendix A.1. Dataset Contents

The Zenodo repository contains four datasets supporting the analyses presented in this study.

#### Appendix A.1.1. Repeated QDM Measurements at Selected Spatial Points

- Nine repeated measurements were acquired at each of sixteen representative spatial points across multiple measurement sessions.
- Measurements were acquired to characterize repeated-measurement variability of the quantum diamond magnetometer (QDM) across multiple field sessions.

#### Appendix A.1.2. Continuous 1-m Interval QDM Survey

- QDM measurements acquired at 1-m spatial intervals along the 90-m test section.
- The dataset covers the asphalt-concrete, approach-slab, plain-concrete, and reinforced-concrete sections.

#### Appendix A.1.3. Raw GPR Data

- Original GPR datasets acquired along A-line and B-line survey paths.
- Three antenna frequencies are provided (250, 500, and 800 MHz).
- Both .rad and .rd3 files are included together with a summary spreadsheet and the Time-Power Gain (TPG) processing script.

#### Appendix A.1.4. Processed Representative GPR Images

- Representative B-scan images generated from the raw GPR datasets.
- Images are provided for asphalt concrete, approach-slab, and reinforced-concrete pavement sections at 250, 500, and 800 MHz.

### Appendix A.2. File Naming Convention

#### Appendix A.2.1. Repeated QDM Dataset

Example: 0508/ascon_B_0m.csv

- Format: [Measurement Date]/[Pavement Type]_[Survey Line]_[Distance].csv
- Distance = local distance from the beginning of the corresponding pavement section (m).
- For the approach-slab files, local distances are converted to the manuscript chainage by adding 30 m; for the concrete-section files, the corresponding manuscript chainage should be interpreted according to the survey layout shown in Figure 1.

where

- Measurement Date = survey date (MMDD format).
- Pavement Type = ascon, approach slab, or reinforced concrete.
- Survey Line = A or B.
- Distance = distance from the survey origin (m).
- Example: 0515/approach slab_A_13m.csv
- Indicates that measurement was taken on 15 May.
- Approach-slab section.
- Survey line A.
- 13 m from the reference origin.

#### Appendix A.2.2. Continuous 1-m Survey

- Example: 44m_approach slab_A.csv
- Format: [Distance]_[Pavement Type]_[Survey Line].csv

where

- Distance = distance from the survey origin (m).
- Pavement Type = ascon, approach slab, or reinforced concrete.
- Survey Line = A.

#### Appendix A.2.3. Raw GPR Dataset

Example: A line 90 m 500 MHz antenna/

- DAT_0072_A1.rad
- DAT_0072_A1.rd3

Each survey consists of

- Survey line (A or B).
- Survey length (90 m).
- Antenna frequency.
- Raw GPR files (.rad and .rd3).

#### Appendix A.2.4. Processed GPR Images

Example: ascon_500 MHz.png

- Format: [Pavement Type]_[Frequency].png

where

- Pavement Type = ascon, approach slab, concrete.
- Frequency = 250, 500, or 800 MHz.
- In the processed GPR image filenames, “concrete” denotes the reinforced-concrete 80–90 m image segment used in Figure 10 and does not refer to the plain-concrete section.
